# The effect of social desirability bias on the measurement of subjective health literacy of children

**DOI:** 10.1093/eurpub/ckac131.346

**Published:** 2022-10-25

**Authors:** T Bollweg, O Okan

**Affiliations:** TUM Department of Sport and Health Sciences, Technical University Munich, Munich, Germany

## Abstract

**Background:**

When measuring children’s health literacy (HL) with self-report questionnaires, there is doubt whether high scores reflect good HL, or if they are caused by social desirability bias, meaning that children present themselves favorably. This study explores how the tendency to answer in a socially desirable way impacts HL scores.

**Methods:**

A cross-sectional study was conducted among fourth-graders in North-Rhine Westphalia, Germany. The study was designed as a representative survey starting in 12/2020, which could not be realized due to pandemic-related constraints. Data collected between 07/21 and 11/21 is included here. Subjective HL was assessed with the HLS-Child-Q15 and a validated 10-item scale (AFS) was used for social desirability bias. Also included in linear regression explaining HL scores are age, sex, country of origin, home language, family affluence (FAS), interest in learning new things about health, the belief that parents are able to answer own questions about health and frequency of conversations with parents about health. Variables were dichotomized where necessary.

**Results:**

n = 364 students are included (49.5% female). Mean age is 9.5 years (SD=.69). In the regression model (F(9, 271)= 6.724, p<.001), which explains 15.5% of variability in HLS-Child-Q15 scores, frequency of communication about health at home (β = .147, p<.01), interest in learning about health (β=.268, p<.001), and the belief that parents are able to answer own questions about health (β =.209, p<.001) are the only significant predictors. Sociodemographic variables and the tendency to answer in a socially desirable way don't have a significant effect on HL.

**Conclusions:**

Our findings suggest that social desirability is not a significant source of bias when measuring children’s HL with the HLS-Child-Q15. Communication about health at home and children’s interest in health seem to be much more important for children’s HL. Further research is necessary to verify these findings.

**Key messages:**

• Social desirability doesn’t seem to be a source of bias when measuring children’s HL with the HLS-Child-Q15.

• Speaking about health at home and children’s interest in health are important predictors of HL.

